# Interrelationship Between Obstructive Sleep Apnea Syndrome and Severe Asthma: From Endo-Phenotype to Clinical Aspects

**DOI:** 10.3389/fmed.2021.640636

**Published:** 2021-06-30

**Authors:** Beatrice Ragnoli, Patrizia Pochetti, Alberto Raie, Mario Malerba

**Affiliations:** ^1^Respiratory Unit, Sant'Andrea Hospital, Vercelli, Italy; ^2^Traslational Medicine Department, University of Eastern Piedmont, Novara, Italy

**Keywords:** sleep-reated breathing disorders, obstructive sleep apnea syndrome, severe asthma, airway inflammation, obesity

## Abstract

Sleep-related breathing disorders (SBDs) are characterized by abnormal respiration during sleep. Obstructive sleep apnea (OSA), a common SBD increasingly recognized by physicians, is characterized by recurrent episodes of partial or complete closure of the upper airway resulting in disturbed breathing during sleep. OSA syndrome (OSAS) is associated with decreased patients' quality of life (QoL) and the presence of significant comorbidities, such as daytime sleepiness. Similarly to what seen for OSAS, the prevalence of asthma has been steadily rising in recent years. Interestingly, severe asthma (SA) patients are also affected by poor sleep quality—often attributed to nocturnal worsening of their asthma—and increased daytime sleepiness and snoring compared to the general population. The fact that such symptoms are also found in OSAS, and that these two conditions share common risk factors, such as obesity, rhinitis, and gastroesophageal reflux, has led many to postulate an association between these two conditions. Specifically, it has been proposed a bidirectional correlation between SA and OSAS, with a mutual negative effect in term of disease severity. According to this model, OSAS not only acts as an independent risk factor of asthma exacerbations, but its co-existence can also worsen asthma symptoms, and the same is true for asthma with respect to OSAS. In this comprehensive review, we summarize past and present studies on the interrelationship between OSAS and SA, from endo-phenotype to clinical aspects, highlighting possible implications for clinical practice and future research directions.

## Introduction

While sleep-related breathing disorders (SBDs) and asthma, two of the most widespread respiratory diseases, have been increasingly well-characterized, their mutual comorbidity and potential bidirectional relationship still need to be fully addressed. Obstructive sleep apnea syndrome (OSAS) is the most common—albeit currently underdiagnosed—form of SBD, with a prevalence of up to 30% in Western populations (1). In the last 20–30 years, there has been growing evidence that OSAS is associated with increased bronchial hyperresponsiveness (2) and inflammation (3) and that may represent an independent risk factor for asthma exacerbations (4). Similarly, the prevalence of asthma has been steadily rising, affecting up to 22% of the population in many countries (5). Many patients suffering from asthma report poor sleep conditions, often attributed to nocturnal worsening of their asthma (6–8), increased daytime sleepiness (6), and a higher prevalence of snoring as compared to the general population (9). Such symptoms are commonly found in OSAS patients, suggesting an association between these two conditions (10). Moreover, these two diseases share common risk factors, such as obesity, rhinitis, and gastroesophageal reflux (GER) (11). However, it still remains to be determined whether—and to what extent—OSAS and asthma are just two distinct diseases with similar symptoms, or they are pathophysiologically associated (10).

## Definitions and Epidemiology

Over the last decade, growing attention has been paid to sleep and SBDs, the latter characterized by abnormal respiration during sleep. According to the International Classification of Sleep Disorders (3rd edition), they can be classified in into four major groups: (i) obstructive sleep apnea (OSA); (ii) central sleep apnea (CSA); (iii) sleep-related hypoventilation; and (iv) sleep-related hypoxemia disorder (12, 13). The most common sleep-related breathing disorder is OSAS, which is increasingly being recognized due to the obesity epidemic and a greater public and physician awareness (14). OSAS is a syndrome characterized by recurrent episodes of partial or complete closure of the upper airway, resulting in disturbed breathing during sleep. The recurrent collapse of the pharyngeal airway during sleep is associated with decreased quality of life (QoL) and significant medical comorbidities (14), leading to apnea or hypopnea and, thus, to intermittent hypoxia and sleep segmentation throughout the night (15–17). Abnormal fluctuations of cardiac rhythm, blood pressure, and intrathoracic pressure are also frequently observed (16, 18). These acute disturbances evolve into mid- and long-term sequelae, such as chronic inflammation, endothelial dysfunction, and metabolic dysregulation (17, 19, 20), followed by hypertension, cardiovascular morbidities (16, 18–20), obesity, insulin resistance (17, 19, 20), impaired cognitive function (16, 20–22), mood, and QoL (16, 20, 22, 23), and premature death (16, 20, 24–26). In recent years, the prevalence of SBDs among the general population has been constantly growing probably due to the availability of more sophisticated and reliable diagnostic devices alongside the aforementioned increased physician awareness. However, these estimates are often biased by methodological issues, such as the different criteria used to define OSAS and the type of technology being employed.

In-laboratory attended diagnostic polysomnography or portable home sleep testing can be used to diagnose sleep apnea (14). In the 80's, an average estimate of prevalence in the general population was set at around 5–30%, with higher rates being more common males (1, 20). In the early 90's, Young and colleagues published the results of the Wisconsin Cohort study (WSCS) (27), the first study on SDB prevalence, confirming that these conditions generally affect more men than women (24 vs. 9%, respectively). The authors also estimated that 2% of women and 4% of men belonging to the middle-aged workforce met the diagnostic criteria for sleep apnea syndrome—i.e., an individual with an apnea-hypopnea score of 5 or higher and co-existing daytime hypersomnolence. These estimates were subsequently revised upwards in a follow-up study (16).

In the following years, new criteria for case definition and revised recommendations for recording and scoring were issued by the American Academy of Sleep Medicine. Furthermore, the combined use of nasal pressure technology and pulse oximeters led to a substantial improvement of the sensitivity of breath recording during sleep hours (20). Thanks to these advances, a large population-based study (HypnoLaus) was able to report a significantly higher prevalence of moderate-to-severe sleep-disordered breathing (≥15 events per h) in men compared to women (49.7 vs. 23.4%, respectively) (20), potentially linked to risk factors such as smoking habits, hormonal influences, and different patterns of fat deposition and responsiveness to inspiratory loading.

Continuous positive airway pressure (C-PAP) therapy is the first-line treatment for OSAS in adults (14). Other modalities include mandibular advancement devices, surgery, or upper airway stimulation therapy. Adjunctive therapy should include weight loss in overweight patients, avoidance of sedatives and alcohol before sleep, and possibly positional therapy (14).

Similarly to what seen for OSAS, also the prevalence of asthma has been steadily rising in recent years, now affecting around 22% of the population in several countries across the globe (5, 28, 29). Indeed, asthma affects more than 330 million people worldwide, and it is likely that by 2025 100 million more people could suffer from it (30, 31). Even though asthma prevalence is higher in developed countries, it continues to rise in low- to middle- income countries where it is associated with a higher mortality rate (31, 32). In the US, the prevalence of asthma in 2017 was 7.9%, with higher rates in children (<18 years; 8.4%) and lower rates in adults (≥18 years; 7.7%) (32). In 2010, nearly 1.8 million emergency room visits were attributed to asthma, and in 2013 this disease accounted for an estimated 10.1 million lost work days among employed adults (33).

To make matter worse, over nine million children in the US have been diagnosed with asthma, of whom 75% have active disease (28, 29). While asthma is more common in males during childhood, it becomes more prevalent in adult women. This gender gap narrows down in the 5th decade, suggesting that sex hormones may play a role in the development of some forms of asthma (31).

Asthma is a multifactorial chronic respiratory disease, usually associated with airway hyperresponsiveness to direct or indirect stimuli, and with persistent airway inflammation. These features may lead to variable expiratory airflow limitation and to the common symptoms of wheeze, shortness of breath, chest tightness, and/or cough (5).

Asthma may have different underlying causes and processes. Indeed, its etiology has been increasingly attributed to interactions between environmental factors (e.g., air pollution, aeroallergens, and weather), host factors (e.g., obesity, nutritional factors, infections, and allergic sensitization), and genetic factors (e.g., asthma susceptibility genes, sex) (31). Interestingly enough, asthma shares a common background of chronic inflammation—and thus statistical association—with other health issues, for example psoriasis and vitiligo (34, 35) or food anaphylaxis (36).

Based on diverse recognizable clusters of demographic elements, clinical features, and pathophysiological characteristics, it is possible to recognize several different “asthma phenotypes.” Some of the common phenotypes indicated by the Global Initiative for Asthma (GINA) 2020 guidelines are summarized in Table 1 (5). Moreover, nearly 3–5% of the total asthma population can be classified as having severe asthma (SA): the small group of SA patients accounts for most of the cost for asthma care (40). The current definition of SA is based on the 2014 ERS/ATS guidelines (41, 42). The diagnostic criteria of SA in patients aged ≥6 years and those of uncontrolled asthma are shown in Table 2.

**Table 1:** Most common phenotypes of asthma (5).

**Allergic asthma**: Associated with a personal history of a respiratory allergen sensitization and, less commonly, with a food, drug, or contact allergy, this phenotype reveals how much dysregulated immunity seems to be important in the development of asthma, with elevated serum immunoglobulin E (IgE) levels, release of mediators from mast cells, skewed T helper 1 (Th1) and Th2 responses and eosinophilic airway inflammation (28), recognizable by the examination of the induced sputum or the evaluation of its surrogate biomarker, the fractional exhaled nitric oxide (FeNO) (37, 38). Often this phenotype responds well to inhaled corticosteroid (ICS) treatment.
**Non-allergic asthma**: The other cluster of patients that do not suffer from allergy; in these patients, the cellular profile of sputum may be neutrophilic, eosinophilic or paucigranulocytic, containing only a few inflammatory cells. ICS therapy has less response in this subgroup of patients.
**Late-onset asthma**: Some patients, women in particular, develop asthma signs and symptoms in adult life for the very first time. Often, this subgroup of patients is non-allergic and requires higher doses of ICS for the correct treatment.
**Asthma with persistent airflow limitation**: This phenotype can develop in adults with long-standing asthma, probably following airway wall remodeling (39).
**Asthma with obesity**: Patients may display strong respiratory symptoms but little eosinophilic airway inflammation.


**Table 2:** Definition of severe asthma (2014 ERS/ATS guidelines).

**Asthma is defined as severe if it was treated with:**
**Asthma which requires treatment with guidelines suggested medications for GINA steps 4–5 asthma (high dose ICS^#^ and LABA or leukotriene modifier/theophylline) for the previous year or systemic CS for ≥50% of the previous year to prevent it from becoming “uncontrolled” or which remains “uncontrolled” despite this therapy**
**Asthma is defined as uncontrolled when it has the following signs and symptoms:**
**a) Poor symptom control**: *ACQ consistently ≥ 1.5, ACT <20 (or “not well-controlled” by NAEPP/GINA guidelines)* b) Frequent severe exacerbations: *Two or more bursts of systemic CS (≥3 days each) in the previous year* c) Serious exacerbations: *At least one hospitalization, ICU stay or mechanical ventilation in the previous year* d) Airflow limitation: *After appropriate bronchodilator withhold FEV1 <80% predicted (despite reduced FEV1/FVC, defined as less than the lower limit of normal)*

# *The definition of high dose inhaled corticosteroids (ICS) is age-specific.*

*GINA, Global Initiative for Asthma; LABA, long-acting β_2_-agonists; CS, corticosteroids; ACQ, Asthma Control Questionnaire; ACT, Asthma Control Test; ICU, Intensive Care unit; NAEPP, National Asthma Education and Prevention Program; FEV1, forced expiratory volume in 1 s*.

A large number of patients suffering from asthma report poor sleep, often attributed to nocturnal worsening of their asthma (6–8), increased daytime sleepiness (6), and a higher prevalence of snoring than that of the general population (9).

## Underlying Pathways

Symptoms present in both OSAS and asthma suggest a possible association between the two conditions. The first demonstration that asthma is associated with an increased risk of developing OSAS came from the Wisconsin Sleep Cohort Study, a randomized population-based prospective study started in 1988, where 547 adult subjects (52% females, mean age 50 years) were subjected to polysomnography every 4 years. Asthma data were recorded by general practitioners or through the administration of questionnaires. Subsequently, the relationship between the incidence of asthma and OSAS and excessive daytime sleepiness (EDS) was examined by regression analysis. The results showed that the relative risk for asthma patients—adjusted by sex, age, and body mass index (BMI)—of developing OSAS during a 4-year observational period was quite high (RR = 1.39). The observation that asthma was significantly related to the development of new OSAS (RR = 2.72, *P* = 0.045) led the authors to conclude that asthma is related to the development of OSAS with EDS (43–45). Further studies suggested a bidirectional correlation between asthma and OSAS, with a mutual negative effect in term of severity. Specifically, OSAS was shown to not just be an independent risk factor for asthma exacerbations (4) but also to worsen asthma (46). Likewise, asthma was shown to exacerbate OSAS in the same study (46). Lastly, the Berlin Questionnaire survey study recorded a more frequent occurrence of daytime sleepiness and apnea in asthmatic patients compared to the general population (10).

The first evidence of a higher frequency of roncopathy in atopic women (47) was described by a 14-year prospective study, showing a pathogenetic role of asthma in sleep respiratory disorder development (48). Other authors evaluated the effect of OSAS on asthma control, reporting that the presence of OSAS was associated with poor asthma control (3) both at daytime and night (11). In addition, OSAS treatment ameliorated asthma symptoms, morning peak flow values, and QoL (12). Further confirmation of the interplay between asthma and OSAS came from a series of polysomnographic studies showing a high OSAS prevalence (up to 90%) in patients suffering from SA (49, 50). Fittingly, findings from an SA cohort study demonstrated that a higher proportion of patients with SA were at high risk of developing OSAS compared to controls (26 vs. 3%, respectively) (51).

According to the results of the Severe Asthma Research Program, SA patients experienced poorer sleep quality, were sleepier, and had a worse QoL than their healthy counterparts. A significant association between the Sleep Apnea Scale of the Sleep Disorders Questionnaire (SA-SDQ) and the count of polymorphonuclear cells in sputum was recorded as well. In particular, each increase in SA-SDQ score by its standard deviation (6.85 units) was associated with a rise in percentage of sputum neutrophils of 7.78 (95% CI 2.33–13.22, *P* = 0.0006), independent of obesity and other confounders (51). Thus, the authors concluded that OSAS symptoms are more prevalent among asthmatics, where they tend to associate with higher disease burden. Finally, among asthma patients, the increased risk of OSAS was associated with the occurrence of neutrophilic airway inflammation, suggesting that OSAS may be an important contributor to neutrophilic asthma (51).

## Patophysiological Correlation Between Obstructive Sleep Apnea Syndrome and Severe Asthma

The observation that OSAS is an independent risk factor of asthma exacerbation and that each condition can worsen the other (51) suggests a bidirectional correlation between asthma and OSAS with a reciprocal negative effect in term of severity. Congruently, SA, SA treatment, and comorbidities can all lead to pharynx alterations, favoring OSAS development. Furthermore, in asthma patients, prolonged inhaled corticosteroid (ICS) therapy in the presence of gastroesophageal reflux is associated with chronic snoring and higher risk of OSAS (52) independently from classical risk factors such as obesity or rhinitis. Finally, long-term ICS therapy can modulate hormones secretion, especially growth hormone release (53, 54), which may cause metabolic and cardiovascular complications (55), thereby worsening the effects of OSAS. The hypothesized mechanism underlying the interrelationship between OSAS and SA is shown in Table 3. More recently, researchers have proposed that OSAS and asthma patients may be more susceptible to SA attacks induced by systemic inflammation (71, 72). These findings strongly suggest that all asthmatic patients should be evaluated for OSAS-associated risk factors, such as gastroesophageal reflux and rhinitis, in order to detect—and eventually treat—sleep apneas. Overall, in light of the higher incidence of respiratory sleep disorders in SA patients, OSAS treatment may reduce the number of asthma attacks, lead to a better control of asthma, and improve the patients' QoL.

**Table 3:** Mechanisms underlying the relationship between asthma and OSAS.

**OSAS (effects on asthma)**
OSAS is linked to increased bronchial hyperresponsiveness (2, 56) and may be an independent risk factor for asthma exacerbations (4).
The presence of OSAS is associated with poor asthma control (43) both at daytime and night (57).
OSAS treatment improves asthma symptoms, morning peak flow values, and QoL (46).
**Asthma (effects on OSAS)**
A higher frequency of roncopathy in atopic women (47) was first reported by a 14-year prospective study showing a pathogenetic role of asthma in sleep respiratory disorder development (48).
Asthma may modify the pharynx collapsibility (58).
During asthma attacks, increased negative intrathoracic pressure may lead to higher pharynx collapsibility (59), which can also arise from decreased respiratory volumes (as in asthma patients) (60).
As asthma is also associated with respiratory muscles weakness and upper ways instability, sleep fragmentation due to asthma with nocturnal symptoms may also induce a loss of slow-waves sleep and cognitive impairment (61).
An additional way from asthma to OSAS is systemic inflammation and use of systemic and inhaled corticosteroids (62, 63). Corticosteroids, frequently prescribed in asthma patients, may lead to a pharyngeal structures remodeling and fatty tissue redistribution (54, 55, 64).
Study on dogs showed that dexamethasone may lead to instability of pharyngeal muscles (“floppy”) reducing their protective effect on upper airways during sleep (65).
**Asthma and obesity**
Major risk factor and a disease modifier of asthma both in children and adults. Obese subjects have increased risk of asthma.
Obese asthmatics have more symptoms, more frequent and severe exacerbations, a reduced response to medications and an overall decreased quality of life (66).
Different phenotypes within the obese asthma syndrome: those seen in lean individuals complicated by obesity, disease newly arising in obese individuals and phenotypes worsens by increased environmental pollutants response (67).
Different factors contributing to the syndrome of obesity-related asthma: Diet, microbiome, genetic factors, metabolic and immune function, environmental exposures, and mechanical effects (68).
**Other conditions and comorbidities**
Toxic effect on the pharyngeal mucosa by gastroesophageal reflux, a frequent condition in asthma (69), associated with laryngospasm and neurogenic inflammation (64).
OSAS patients display a higher frequency of acid reflux along with micro-aspiration phenomenon of gastric acid and nocturnal bronchoconstriction (70), thereby establishing a bidirectional relationship.

*OSAS, Obstructive Sleep Disorder Syndrome; QoL, Quality of Life*.

## Asthma Involvment in the Pathogenesis of Obstructive Sleep Apnea Syndrome

Different observations highlighted a relationship between asthma symptoms and their impact on sleep quality revealing often a coexistence of asthma and obstructive sleep apnea syndrome. Risk factors common to the two diseases include obesity, rhinitis and gastroesophageal reflux. Airway and systemic inflammation, neuromechanical effects of recurrent upper airways collapse and asthma medications are additional explanatory factors. Recent evidences demonstrated the underlying mechanisms to the development of OSAS in asthma patients. An interesting research by Kalra et al. in a large population-based cohort of young atopic women found 20.5% prevalence of habitual snoring (≥3 nights per week), moreover they also reported a significant association between asthma and snoring. This effect was independent of upper respiratory tract symptoms (e.g., rhinitis), cigarette smoking, and race (47). Another longitudinal 14-year prospective study on risk factors for habitual snoring in a general adult population indicated the presence of asthma as a risk factor for snoring, showing its pathogenetic role for sleep-disordered breathing development (48). It was previously observed how asthma may modify the pharynx collapsibility (58), in particular during asthma attacks, increased negative intrathoracic pressure may lead to higher pharynx collapsibility (59), which can also arise from decreased respiratory volumes (the same happens in asthma patients) (60). As asthma is also associated with respiratory muscles weakness and upper ways instability, sleep fragmentation caused by asthma nocturnal symptoms may also induce a loss of slow-waves sleep and cognitive impairment (61) An additional link between asthma and OSAS is sustained by systemic inflammation and the use of systemic and inhaled corticosteroids (62, 63). Corticosteroids, frequently prescribed in asthma patients, may lead to a pharyngeal structures remodeling (64). Study on dogs showed that dexamethasone may lead to instability of pharyngeal muscles (“floppy”) reducing their protective effect on upper airways during sleep (65). Lastly it is important to underline the toxic effect on the pharyngeal mucosa by gastroesophageal reflux, a frequent condition in asthma (69), associated with laryngospasm and neurogenic inflammation (64). OSAS patients display a higher frequency of acid reflux along with micro-aspiration phenomenon of gastric acid and nocturnal bronchoconstriction, thereby establishing a bidirectional relationship (70).

## Relationship Between Asthma and Obesity

Obesity represents a major risk factor and a disease modifier of asthma both in children and adults (66). It was found that obese subjects have increased risk of asthma and obese asthmatics have more symptoms, more frequent and severe exacerbations, a reduced response to medications and an overall decreased quality of life highlighting a bidirectional correlation between these two entities. There are likely different phenotypes within the obese asthma syndrome: those seen in lean individuals complicated by obesity, disease newly arising in obese individuals and phenotypes worsens by increased environmental pollutants response (67). Different factors contributing to the syndrome of obesity-related asthma have been hypothesized such as diet, the microbiome, genetic factors, metabolic and immune function (oxidative stress, cytokines, innate, and adaptive immunity), environmental exposures and mechanical effects: reduction in functional residual capacity (FRC) and expiratory reserve volume (ERV) (68). As obese asthma syndrome is a complex and multifactorial entity which is just beginning to be understood further studies should better characterize this disease to understand, in particular, mechanisms conducting to the phenotype of severe and uncontrolled asthma.

## Clinical Implications

**Figure 1:**
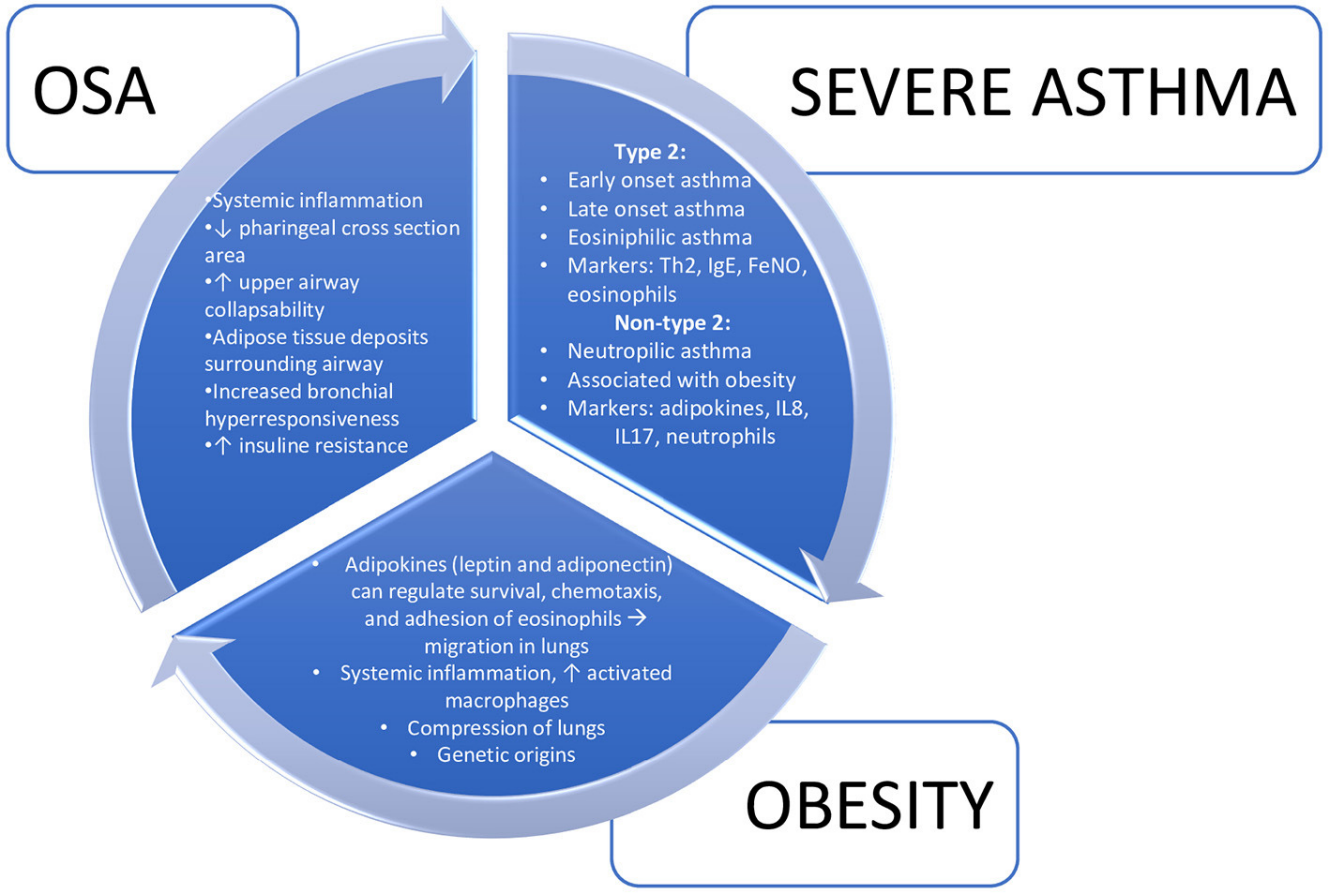
Interrelationships between severe asthma, obesity, and obstructive sleep apnea.

It remains unclear whether OSAS in asthmatic subjects is merely a comorbidity or a specific “subphenotype” of asthma. On the one hand, patients with allergic asthma are often characterized by T2-driven inflammation and over-production of IL-5—resulting in airway eosinophilia—and IL-13—leading to airway smooth muscle hyperresponsiveness and mucus hypersecretion—, complicated by the development of obesity and OSAS (40, 73). On the other hand, in obese female patients with later onset of non-allergic asthma, mechanical changes affecting the lung function (i.e., restrictive pattern) may favor obstructive apnea development. In these patients, adipose tissue are known to secret several cytokines and adipokines that can have a direct effects on the airway epithelium and trigger bronchial hyperreactivity (Figure 1) (66, 74).

The relationship between OSAS, obesity, and asthma may be even more complex and may involve other players, including mechanical, dietary, and genetic factors (75). A plausible explanation to this interplay comes from the “integrated airway” hypothesis, according to which an inflammatory process within a continuous upper airway obstruction results in intrathoracic pressure swings, frequent arousals, and intermittent hypoxia, all contributing to an inflammatory milieu, as demonstrated by the association of OSAS with cardiovascular and cerebrovascular diseases (40, 76). The result of the association of OSAS with SA is an increased risk of comorbidities, such as cardiovascular sequelae (51).

For the reasons above mentioned polysomnography is recommended in asthmatics with inadequate control of nocturnal symptoms despite adequate treatment (77).

## How OSAS Therapy Can Affect Asthma and Viceversa

A recent research has investigated the relationship between OSAS, asthma and quality of life, going deeply on the prevalence of sleep impairment and predictors of sleep quality among asthmatic patients (78). The results of this study highlight that sleep disturbance is a common problem among these patients and suggest that sleep quality can be predicted considering the level of asthma control and the presence of comorbid conditions. The results of this study suggest the need for clinicians to detect poor sleep quality in these subjects to better address the correct and targeted treatment for each condition. Previous studies have shown, for example, that PAP treatment is beneficial to control sleep disturbances either in OSAS and asthmatic patients. In asthmatic patients with OSAS, C-PAP treatment may lead to a significant improvement in asthma symptoms, morning peak expiratory flow, and quality of life (QoL) (79). Fittingly, C-PAP treatment reduced asthma symptoms and bronchodilator use while improving lung function tests and QoL (40). CPAP in this context represent a valid tool to integrate with the corticosteroid treatment considering that corticosteroids, may have side effects like leading to a pharyngeal structures remodeling, fatty tissue redistribution, impairment of growth hormone (GH)-insulin-like growth factor (IGF)-1 axis conducting to a negative role in bone metabolism (54, 55, 64). Moreover, studies on animals demonstrated that corticosteroids may enhance force fluctuation-induced relengthening in contracted airway smooth muscle leading to instability of pharyngeal muscles reducing their protective effect on upper airways during sleep (65) and conducting to a worsening of OSAS.

A prospective trial by Serrano-Pariente et al. has shown that the proportion of adult asthmatic patients suffering from uncontrolled asthma decreased from 41.4 to 17.2% in response to C-PAP therapy. Likewise, the percentage of patients experiencing asthma attacks during the course of 6 months decreased from 35.4 to 17.2% following C-PAP treatment (80).

In good agreement, OSAS treatment in SA patients has been shown to influence the cardiovascular risk. Oxidative stress and systemic inflammation may, in fact, lead to metabolic dysregulation in OSAS patients (44, 46).

The fact that OSAS patients, regardless of their weight, display increased oxidative stress with vascular endothelial dysfunction has led to the hypothesis that continuous positive airway pressure therapy (C-PAP) and anti-oxidant treatment (vitamin C) may improve endothelial function (68). Moreover, in OSAS patients, atherosclerosis symptoms can be ameliorated through mandibular advancement devices (MADs) or C-PAP (81), suggesting that OSAS treatment, primitive or comorbid, may prevent cardiovascular sequelae, such as acute myocardial infarction, stroke, and acute fatal cardiovascular events.

## Conclusions and Future Directions

In conclusion, mounting evidence appear to indicate that there exists a relationship between OSAS and SA based on shared pathophysiological factors and bidirectional interactions (40). Similarly to asthma, OSAS promotes inflammatory responses by means of hypoxia, hypercapnia, and sleep fragmentation, resulting in a reversible increase in C-reactive protein (CRP). Production of TNF-α, a pro-inflammatory cytokine, is elevated in OSAS patients and plays an important role in collapse and re-opening of the airways. Both pro-inflammatory factors tend to decrease following C-PAP treatment (77), thereby improving asthma symptoms and QoL (40). In our experience, C-PAP therapy can also improve lung function tests in adult asthmatic patients (unpublished data).

Taken all together, the results from the literature highlight the importance of assessing the co-existence of asthma, chiefly SA—a condition with poor prognosis and higher managements cost—, in OSAS patients as well as the presence of other sleep-related breathing disorders and apneas, especially when asthma is difficult to control or comorbid.

Overall, the key observation that OSAS treatment reduces cardiovascular sequelae while improving the QoL (11) points to the urgent need to fill critical gaps in our knowledge about endotypes, phenotypes, and comorbidities in OSAS.

## Author Contributions

MM conceived the idea of the manuscript. BR, AR, and PP contributed to various parts of the text, wrote the manuscript, and prepared the tables and bibliography. All the authors edited, revised, commented on the manuscript, and approved the submitted version of the article.

## Conflict of Interest

The authors declare that the research was conducted in the absence of any commercial or financial relationships that could be construed as a potential conflict of interest.
